# Mechanistic insights into the role of oral microbiome in the malignant transformation of oral lichen planus to oral squamous cell carcinoma

**DOI:** 10.3389/fonc.2026.1694005

**Published:** 2026-04-07

**Authors:** Yue Wu, Yaoqi Chen, Jingjing Mao, Kelong Li, Qiao He, Zhongwei Zhou

**Affiliations:** 1 College of Stomatology, Ningxia Medical University, Yinchuan, Ningxia, China; 2 Ningxia Province Key Laboratory of Oral Diseases Research, Yinchuan, Ningxia, China; 3 Stomatological Hospital, General Hospital of Ningxia Medical University, Yinchuan, Ningxia, China; 4 Department of Oral and Maxillofacial Surgery, General Hospital of Ningxia Medical University, Yinchuan, Ningxia, China

**Keywords:** epithelial-mesenchymal transition, malignant transformation, oral lichen planus, oral microbiome, oral squamous cell carcinoma

## Abstract

Oral Lichen Planus (OLP) is a common oral potentially malignant disorder, and its transformation into Oral Squamous Cell Carcinoma (OSCC) has become a research focus. In recent years, increasing attention has been paid to the role of the oral microbiome in tumor initiation and progression. Studies have shown that dysbiosis of the oral microbiome may contribute to and accelerate the malignant transformation of OLP to OSCC through multiple mechanisms, including the induction of inflammatory responses, disruption of immune regulation, promotion of oxidative stress, and epithelial-mesenchymal transition (EMT). This review summarizes recent advances in research on the characteristic changes in the oral microbiome and associated molecular mechanisms during the malignant transformation of OLP, aiming to provide a theoretical basis and scientific support for early warning and microecological-targeted interventions in OLP malignancy.

## 1 Introduction

Oral lichen planus (OLP) is a chronic inflammatory, T-cell-mediated disease of the oral mucosa, with a global prevalence ranging from 0.1% to 4.0%, predominantly affecting middle-aged women. The World Health Organization has classified OLP as one of the oral potentially malignant disorders (OPMDs) (1). Oral squamous cell carcinoma (OSCC), the most common malignancy of the head and neck region, accounts for approximately 90% of all oral cancer cases (2). Patients with OPMDs are at a higher risk of developing invasive oral cancer compared to individuals with healthy oral mucosa (3). Therefore, the malignant transformation risk of OLP has drawn considerable attention. Meta-analyses evaluating the malignant potential of OLP and related lesions have reported that about 1.43% of OLP cases eventually progress to OSCC (4). In light of this risk, the Chinese “Guidelines for the Diagnosis and Treatment of Oral Lichen Planus (2022 Edition)” recommend regular follow-up (every 3–6 months) for OLP patients, especially those with high-risk subtypes, to enable early detection of potential malignant transformation (5).

In recent years, the pivotal role of the oral microbiome in maintaining both local and systemic health has become increasingly evident. This homeostasis relies on a complex and finely tuned interaction between the host and diverse microbial communities within the oral cavity (6). Disruption of this balance, known as dysbiosis, may contribute to the development of various oral and systemic diseases (7). The occurrence of OSCC is associated with multiple molecular alterations, such as p53 mutations, epigenetic modifications, and immune dysregulation, while chronic inflammatory stimulation can lead to mucosal barrier disruption, apoptosis, DNA damage accumulation, and aberrant cell proliferation, thereby creating a permissive environment for OLP malignant transformation (8, 9). Consequently, the link between OLP and OSCC lies in their shared inflammatory microenvironment and underlying molecular pathways.

Against this backdrop, this review focuses on alterations in the oral microbiome during the malignant transformation of OLP and explores their potential carcinogenic mechanisms.

## 2 Dysbiosis of oral microbiome in OLP and OSCC

### 2.1 Microbial changes in OLP

**Figure 1:**
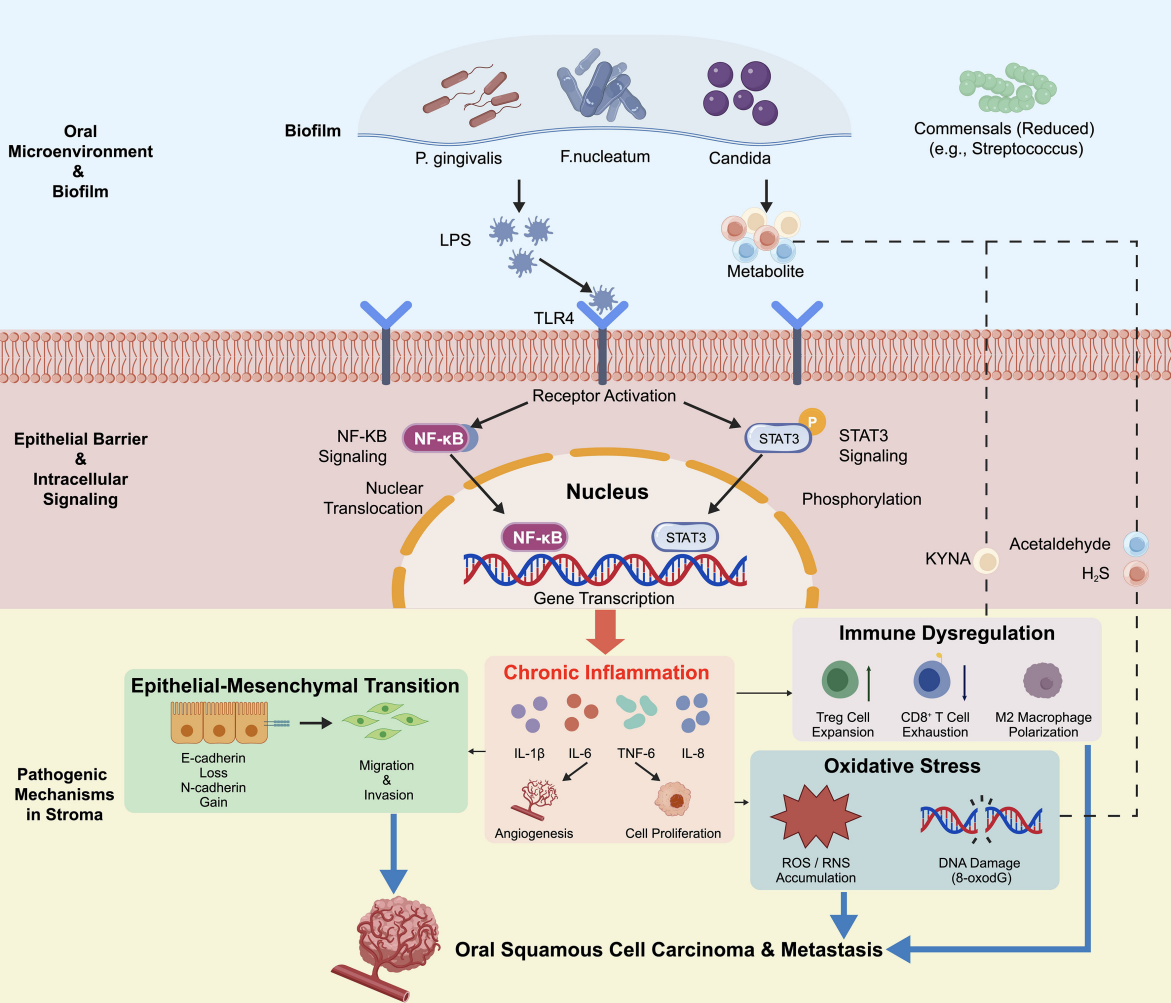
Schematic diagram illustrating the molecular mechanisms by which oral dysbiosis drives the initiation and metastasis of oral squamous cell carcinoma. The diagram was created with BioGDP (Jiang et al., 2025), a generic diagramming platform for biomedical graphics (available at: https://biogdp.com) (70). 1. Oral microenvironment and biofilms: Enrichment of pathobionts (*P. gingivalis*, *F. nucleatum*, *C. albicans*) and depletion of commensals (*Streptococcus* spp.), releasing LPS, acetaldehyde, kynurenic acid, and H_2_S. 2. Epithelial signaling: Metabolites activate TLR4, NF-κB, and STAT3, promoting nuclear translocation. 3. Stromal mechanisms: Immune dysregulation (Treg expansion, CD8^+^ T cell exhaustion, M2 polarization), chronic inflammation (IL-1β/6/8, TNF-α), EMT (loss of E-cadherin, gain of N-cadherin, increased invasion), oxidative stress (ROS/RNS, 8-oxo-dG). 4. Outcomes: Stromal angiogenesis, proliferation, malignant transformation, metastasis. 5. Symbols: Solid arrow →, promotion; T-bar ⊣, inhibition; dashed arrow –––, transmembrane transport.

16S ribosomal RNA (16S rRNA) sequencing and metagenomic sequencing are currently the primary approaches for investigating the oral microbiome. 16S rRNA sequencing enables rapid and cost-effective analysis of microbial composition, making it suitable for large-scale comparisons of microbial diversity and abundance, particularly for taxonomic identification of bacterial communities. Metagenomic sequencing, which does not rely on amplification of specific genes, directly sequences all microbial DNA in a sample at high throughput, allowing analysis of functional genes, metabolic pathways, and resistance genes within microbial communities, thereby revealing potential functional differences between health and disease. By applying these two techniques to samples from healthy individuals, OLP patients, and OSCC patients, significant differences in microbial composition and functional metabolic pathways can be identified (**Table 1**, **Figure 1**), providing important insights into the pathogenesis of OPMDs and potential targets for microbiome -based interventions.

**Table 1:** Dynamics of the oral microbiome across healthy state, oral lichen planus, and oral squamous cell carcinoma.

Disease stage	Major bacterial profile changes	Direction of change	Key cariogenic/oncogenic genera and metabolites
Healthy	*Streptococcus, Actinomyces, Neisseria, Rothia*	*Baseline*	*-*
Early OLP	*Prevotella, Solobacterium, Corynebacterium*	*Partial decline / Slight increase*	*Prevotella melaninogenica (cytotoxin-producing)*
Long-standing OLP	*Actinomycetia, Streptococcus, Porphyromonas gingivalis*	*Marked increase in abundance*	*Porphyromonas gingivalis Metabolites: LPS, gingipains*
Early OSCC	*Fusobacterium nucleatum, Prevotella intermedia, Streptococcus, Actinomycetia*	*Marked increase in abundance*	*Fusobacterium nucleatum Metabolites: FadA adhesin, extracellular vesicles (EVs)*
Long-standing OSCC	*Fusobacterium, Peptostreptococcus, Clostridia*	*Marked increase in abundance*	*Fusobacterium Metabolites:butyrate*

This table systematically summarizes the compositional evolution of the oral microbial community across different clinical stages, ranging from a healthy state to oral lichen planus (OLP) and further to oral squamous cell carcinoma (OSCC). By comparing the major shifts in bacterial composition, the direction of these changes, and the key pathogenic genera along with their metabolites at each stage, this table illustrates the dynamic process of microbial dysbiosis during the malignant transformation from OLP to OSCC. It highlights a progressive shift from a relatively balanced microbial community towards severe dysbiosis: health-associated commensals (e.g., *Streptococcus*, *Neisseria*) gradually decrease, while opportunistic pathogens with pro-inflammatory and pro-carcinogenic potential (e.g., *Porphyromonas gingivalis*, *Fusobacterium nucleatum*) and their associated virulence factors (e.g., LPS, butyrate, FadA adhesin) become significantly enriched. This provides a visual map of microbial succession for understanding the driving role of the oral microbiome in OLP malignancy.

A 2023 study from Yenepoya University compared the oral microbiome of healthy controls and OLP patients, revealing that the control group was dominated by commensal bacteria such as *Streptococcus*, *Neisseria*, and *Veillonella*. In contrast, OLP patients exhibited a significant reduction in commensals, including *Actinomyces* and *Corynebacterium*, while potential pathogens such as *Mycoplasma* and *Capnocytophaga* were significantly enriched. Notably, the detection rate of *Candida* in erosive OLP patients reached 60%. Regarding microbial diversity, the Chao1 index in the OLP group was significantly decreased (p = 0.014), indicating reduced richness, and β-diversity analysis revealed a significant structural difference between OLP patients and healthy individuals (p = 0.038) (10).

### 2.2 Microbial changes in OSCC

Recent studies have further confirmed that the oral microbiome in OSCC patients exhibits marked dysbiosis, characterized by pathogen enrichment and commensal depletion. A 2025 study published in *Clinical Oral Investigations* compared the oral microbiome of healthy individuals and OSCC patients, showing a typical dysbiotic pattern in OSCC: health-associated commensals such as *Streptococcus*, *Neisseria*, and *Rothia* were significantly reduced, whereas potential pathogens including *Fusobacterium nucleatum* (Fn), *Porphyromonas gingivalis* (Pg), and *Leptotrichia* were enriched. The oral microbiome diversity was also significantly decreased and negatively correlated with tumor T stage, suggesting that dysbiosis may contribute to tumor progression (11).

Additionally, Koketsu et al. conducted stage-specific analyses and revealed dynamic changes in the oral microbiome across OSCC progression. Compared with healthy controls, early-stage OSCC patients exhibited an increase in *Streptococcus* and Actinobacteria in saliva, whereas late-stage OSCC patients showed marked enrichment of pathogens, including *Fusobacterium*, *Dialister*, and Clostridia (12).

Mechanistically, Fn suppresses the cGAS–IFN-β pathway through succinate metabolism, thereby limiting CD8^+^ T cell infiltration and weakening antitumor immunity; Pg synergizes with Fn to promote OSCC cell cycle progression and IL-6/IL-8 secretion, exacerbating the inflammatory microenvironment (13). Moreover, Prevotella intermedia can co-aggregate with Pg and Fn to form the “red complex,” potentially exerting pro-carcinogenic effects during chronic inflammation and malignant transformation (14). Koketsu et al. further suggested that the late-stage enrichment of Dialister and Clostridia may contribute to tumor progression through amino acid metabolism or cholesterol biosynthesis pathways, warranting further functional investigation (12).

## 3 Key pathogenic mechanisms of oral microbiome dysbiosis in promoting malignant transformation from OLP to OSCC

### 3.1 Chronic inflammation-mediated malignant transformation

Bacterial metabolites and surface components, such as lipopolysaccharides (LPS), lipoteichoic acids (LTA), flagellin, and CpG DNA, act as pathogen-associated molecular patterns (PAMPs) that are recognized by host pattern recognition receptors (PRRs), including Toll-like receptors (TLRs) and NOD-like receptors (NLRs). This recognition triggers multiple downstream signaling pathways. For example, TLR4-mediated signaling promotes the activation of nuclear factor κB (NF-κB), leading to the production of pro-inflammatory cytokines such as interleukin-1β (IL-1β), interleukin-6 (IL-6), and tumor necrosis factor-α (TNF-α). Concurrently, the assembly and activation of the NOD-like receptor pyrin domain-containing protein 3 (NLRP3) inflammasome activate caspase-1, facilitating the maturation and secretion of IL-1β and interleukin-18. These signaling cascades collectively induce sustained release of pro-inflammatory cytokines, initiating and maintaining chronic inflammation. Various inflammatory mediators have been implicated in OSCC initiation and progression, including the NF-κB and AP-1 pathways, TNF-α, IL-6/IL-8, and members of the IL-1 family (15). Bacteria can trigger persistent inflammation through activation of TLR/NF-κB and IL-6/STAT3 pathways, promoting cell proliferation and shaping the tumor microenvironment.

NF-κB is a central inflammatory transcription factor commonly expressed in tumors, regulating genes involved in inflammation, cell proliferation, tumorigenesis, and survival (16–18). It is typically activated by TNF-α, IL-1, and LPS (17). Simultaneously, NF-κB and STAT3 form a central signaling hub in inflammation-associated tumorigenesis. Their sustained activation not only maintains chronic inflammatory signaling but also contributes to the remodeling of the tumor microenvironment (TME).Persistent NF-κB activation can upregulate programmed death-ligand 1 (PD-L1) expression, suppress T cell activation and cytotoxicity, and facilitate immune evasion, thereby creating an immunosuppressive tumor microenvironment conducive to tumor development and progression. In chronic inflammatory states, such as colitis or OLP, IL-6 can further enhance NF-κB activity via phosphorylation of STAT3 (p-STAT3), forming a positive feedback loop between inflammation and carcinogenesis (IL-6 amplification). Moreover, STAT3 activation promotes M2 macrophage polarization and upregulates vascular endothelial growth factor (VEGF), thereby enhancing angiogenesis, metastasis, and immune escape, contributing to malignant progression (19).

TNF-α is a multifunctional cytokine recognized as a key mediator in cancer development (20). It can induce epithelial-mesenchymal transition (EMT) (21), promote angiogenesis (22), and enhance tumor invasiveness (23). TNF-α is also expressed in OLP (24), where it regulates genes associated with neutrophil recruitment, invadopodia formation, and tumor invasion.

IL-6 and IL-8, members of the interleukin family, are prototypical pro-inflammatory cytokines that activate downstream pathways via their respective receptors IL-6R and IL-8R. IL-6 participates in cell proliferation through RAS/RAF/MAPK, Akt/PI3K/mTOR, STATs, and Src/YAP/Notch pathways, regulates survival, metabolism, and oxidative stress via Akt/PI3K/mTOR and STAT signaling, and contributes to tissue morphogenesis via Src/YAP/Notch signaling (25). IL-6 also regulates migration, angiogenesis, differentiation, and immune modulation (26). IL-8 has been shown to induce proliferation, angiogenesis, invasion, chemotaxis, and apoptosis regulation in various cancers via STAT3/NF-κB signaling (27–29). Both IL-6 and IL-8 possess chemotactic activity, recruiting neutrophils and monocytes to inflamed tissues (30, 31).

In summary, chronic inflammation-mediated malignant transformation is a complex, multi-factorial, multi-pathway, and multi-stage process. Bacterial infection acts as the initial trigger, with PAMPs recognized by host PRRs, activating core signaling pathways such as TLR/NF-κB and NLRP3 inflammasome, leading to sustained release of pro-inflammatory cytokines including TNF-α, IL-6, and IL-8. These signaling events not only sustain chronic inflammatory activity but also form an interconnected regulatory network through pathways such as STAT3, MAPK, and PI3K/Akt. This network promotes a series of downstream oncogenic processes, including oxidative stress, immune dysregulation, and epithelial–mesenchymal transition. Consequently, chronic inflammation can be regarded as a key molecular starting point linking oral microbiota dysbiosis to malignant transformation.

### 3.2 Oxidative stress and DNA damage

Oxidative stress is considered a critical molecular event in the malignant transformation of oral potentially malignant disorders (OPMDs) to oral squamous cell carcinoma (OSCC), primarily mediated by the accumulation of reactive oxygen species (ROS) and reactive nitrogen species (RNS), leading to DNA damage and genomic instability that drive tumorigenesis (32–34). As byproducts of cellular metabolism and inflammatory responses, when the production of ROS and RNS exceeds the host antioxidant capacity results in a state of oxidative stress. ROS can react directly with guanine to form 8-oxo-7,8-dihydro-2′-deoxyguanosine (8-oxodG), a widely recognized biomarker of oxidative DNA damage. During DNA replication, 8-oxodG can induce G:C→T:A transversion mutations; if these occur in key tumor suppressor genes (e.g., *p53*) or proto-oncogenes (e.g., *Ras*), they may initiate malignant transformation.

Simultaneously, inflammatory cells generate excessive nitric oxide (NO) via inducible nitric oxide synthase, producing various reactive nitrogen species, including peroxynitrite (ONOO^−^) and other nitrosative molecules. ONOO^−^ acts as both a strong oxidant and a potent nitrating agent, capable of reacting with guanine to form 8-nitroguanine. This product is chemically unstable and readily depurinates, creating abasic sites that lead to base deletions or misrepair, thus promoting gene mutations and serving as a biomarker of nitrative DNA damage. Importantly, this process occurs not only within inflammatory cells but also within epithelial cells, indicating that the inflammatory microenvironment can directly affect target cells via paracrine signaling, increasing their DNA damage load and malignant transformation risk. This mechanism may help explain the link between OLP and OSCC, where chronic inflammation induces oxidative and nitrative DNA damage in epithelial cells, bridging inflammation and carcinogenesis at the molecular level (35).

Beyond host inflammatory responses, microbial metabolites from specific oral pathogens are a significant source of oxidative stress. *Fusobacterium nucleatum*, a core pathogen associated with periodontitis and oral cancer, can produce hydrogen sulfide (H_2_S), a nucleophilic gaseous toxin. H_2_S mediates oxidative stress primarily by targeting the host antioxidant enzyme system. Its strong reducing properties enable it to chelate metal ions (e.g., Zn^2+^, Cu^2+^) at the active centers of superoxide dismutase (SOD) or modify critical cysteine residues via sulfhydration, resulting in substantial loss of SOD activity (36). Experimental data in HepG2 cell models demonstrate that H_2_S from *F. nucleatum* significantly increases 8-hydroxy-2′-deoxyguanosine (8-OHdG) levels (p < 0.01), a widely used marker of DNA oxidative damage reflecting ROS-induced nuclear and mitochondrial DNA injury. These findings suggest that H_2_S from *F. nucleatum* may promote carcinogenesis in the oral mucosa via oxidative stress, playing a potential regulatory role in OPMD malignant transformation under dysbiotic conditions (37).

Moreover, volatile sulfur compounds (VSCs) produced by pathogenic oral bacteria, such as dimethyl sulfide and H_2_S, can directly damage cellular structures. Their small molecular size and lipophilicity allow them to penetrate cell membranes and target mitochondrial electron transport chains, inhibiting cytochrome c oxidase, inducing mitochondrial dysfunction, and promoting excessive accumulation of superoxide anion (O_2_^−^) and hydrogen peroxide (H_2_O_2_). O_2_^−^, as a primary ROS, can dismutate to H_2_O_2_, which in the presence of metal ions is further converted into highly reactive hydroxyl radicals that directly attack the DNA backbone, causing single- and double-strand breaks and compromising genomic integrity.

Concurrently, ROS serve as secondary messengers, activating multiple inflammation-related signaling pathways, including NF-κB and mitogen-activated protein kinase (MAPK) pathways. NF-κB activation promotes transcription of downstream pro-inflammatory cytokines, such as TNF-α, IL-6, and IL-8, further recruiting inflammatory cells and establishing a positive feedback loop between inflammation and oxidative stress. Persistent MAPK activation regulates cell proliferation, differentiation, and apoptosis, inhibiting normal cell death and allowing survival and clonal expansion of DNA-damaged cells. Collectively, excessive ROS accumulation drives OPMD cell malignant transformation via dual mechanisms: direct DNA damage and indirect modulation of pro-inflammatory signaling (38). These signaling axes have been demonstrated in the aforementioned chronic inflammatory processes to function as key molecular nodes linking microbial stimulation to tumorigenesis. Accordingly, oxidative stress not only promotes genetic mutations through direct induction of DNA damage but can also amplify inflammatory responses by modulating inflammation-related signaling networks, thereby forming a molecular bridge between chronic inflammation and tumor development.

### 3.3 Host immune dysregulation

These changes suggest that, under the combined influence of persistent inflammatory stimuli and microbial dysbiosis, the local immune regulatory network gradually shifts from an “immune defensive” state to an “immunosuppressive” state, thereby creating a microenvironment conducive to tumor cell proliferation and immune evasion. Oral lichen planus (OLP) is characterized histologically by a band-like T cell infiltration, indicating a strong association with immune dysfunction, and autoimmune mechanisms are thought to play a central role in its pathogenesis. Regulatory T cells (Tregs), as core mediators of immune tolerance, secrete immunosuppressive cytokines such as IL-10 and TGF-β, negatively regulating the activation and proliferation of effector T cells (39). Compared with healthy controls, both lesional tissues and peripheral blood of OLP patients exhibit increased numbers of Tregs, suggesting that the dynamic balance among different lymphocyte subsets influences disease progression. This elevation likely reflects a compensatory negative feedback mechanism during chronic inflammation, aimed at limiting excessive immune-mediated tissue damage. However, functional defects or abnormal distribution of Tregs may impair their ability to suppress local effector T cell activity, leading to immune imbalance. Further analyses of OLP lesions indicate a negative correlation between Treg abundance and disease activity, i.e., lower Treg infiltration is associated with more active lesions, suggesting that Treg insufficiency or dysfunction contributes to persistent disease progression. Notably, immunosuppressive therapy significantly increases peripheral Treg proportions in OLP patients (40), indicating that such interventions may restore Treg number or function, re-establish immune tolerance, and alleviate clinical symptoms.

Emerging evidence also highlights the role of gut-derived microbiome in modulating Treg differentiation and function. In OLP patients, short-chain fatty acid (SCFA)-producing bacteria such as *Faecalibacterium prausnitzii*, *Bifidobacterium adolescentis*, and *Bifidobacterium longum* are significantly reduced. This dysbiosis limits SCFA production, impairs Treg differentiation and function, and disrupts local oral immune tolerance, allowing aberrant T cell responses to persist, ultimately promoting OLP initiation and progression (39).

In recent years, increasing evidence suggests that the oral microbiome not only participates in OSCC development via inflammation but also actively shapes the immune microenvironment through metabolite production. Specific microbial metabolites act as molecular mediators that directly modulate host immune cell function, thereby facilitating carcinogenesis. This conceptual shift elevates the oral microbiome from a passive participant in inflammation to an active “metabolic organ” regulating host immunity, providing a novel framework to understand the link between dysbiosis and malignant transformation.

Dysbiotic oral microbiome and tumor-associated colonization by specific bacteria, such as *Streptococcus mutans*, may enhance production of tumor-promoting metabolites, exacerbate oral mucosal carcinogenesis, reprogram a highly immunosuppressive tumor microenvironment (TME), and promote OSCC (41). Kynurenic acid (KYNA), the end product of the tryptophan metabolism pathway, has recently been recognized as an immunosuppressive metabolite. KYNA modulates immune cell differentiation and function primarily via activation of the aryl hydrocarbon receptor (AhR) and G protein-coupled receptor 35 (GPR35). Specifically, KYNA induces T cell quiescence and preferentially promotes differentiation of naïve CD4^+^ T cells into Foxp3^+^ Tregs, thereby altering the effector T cell/Treg ratio and creating an immunosuppressive microenvironment (42, 43).

Mechanistic studies further demonstrate that excessive KYNA production can profoundly remodel the TME: it upregulates KYNA transporters on neutrophils, enhances uptake of KYNA, promotes synthesis and release of S100a8/S100a9 (calcium-binding proteins with dual pro-inflammatory and immunosuppressive functions), increases neutrophil expansion and infiltration within the TME, reduces CD8^+^ T cell infiltration, and elevates local IL-1β levels. IL-1β not only promotes inflammation but also induces CD8^+^ T cell exhaustion, characterized by upregulation of inhibitory receptors (e.g., PD-1, Tim-3) and reduced secretion of effector cytokines (IFN-γ, TNF-α) (41). Thus, KYNA acts as a critical metabolic driver of tumor microenvironment reprogramming, promoting a highly immunosuppressive milieu by inhibiting antitumor immunity, facilitating the infiltration of immunosuppressive cells, and inducing CD8^+^ T cell exhaustion. This finding provides a novel mechanism by which oral microbial metabolites directly modulate immune cells and reshape the TME to promote carcinogenesis.

Oral dysbiosis can profoundly disrupt local and systemic immune homeostasis, weakening antitumor immune surveillance. Changes in microbial composition affect mucosal immunity locally and can, through metabolite production or bacterial translocation, remotely influence systemic immune status. Dysbiosis has been shown to increase Treg numbers and disturb Th17/Treg balance, diminishing host immune surveillance. Th17 cells, as pro-inflammatory effectors, have dual roles in antitumor immunity, while Tregs suppress effector responses; imbalance between these subsets is a critical mechanism of immune evasion. A study in *Nature Communications* revealed a molecular mechanism whereby oral bacteria regulate Th17/Treg homeostasis. *Porphyromonas gingivalis* (Pg), a key periodontal pathogen, can transit via swallowed saliva to the gut, altering gut microbiome composition, disrupting barrier integrity, and promoting entry of bacterial metabolites (e.g., LPS, SCFAs) into systemic circulation. These metabolites modulate T cell differentiation in distal lymphoid organs, perturbing the Th17/Treg balance, and ultimately facilitate OSCC development (44). This work links local oral infection with systemic immune remodeling and distal tumorigenesis, providing a macro-level “oral–gut–immune” axis for understanding the role of oral microbiome in oral carcinogenesis.

### 3.4 Epithelial-mesenchymal transition and dysregulated apoptosis

Epithelial-mesenchymal transition (EMT) is a critical mechanism underlying tumor invasion and metastasis, promoting cancer cell migration and invasiveness by altering cell adhesion and motility. In the development of oral squamous cell carcinoma (OSCC), microbial dysbiosis is increasingly recognized as a key driver of EMT. Dysbiotic microbiome not only regulates EMT-related gene expression but also interferes with programmed cell death and modulates the immune microenvironment, collectively enhancing tumor invasiveness. Recent studies have indicated that oral microbial dysbiosis contributes to tumorigenesis not only through chronic inflammation and oxidative stress but also by driving epithelial–mesenchymal transition (EMT), thereby facilitating tumor invasion and metastasis. This process typically occurs against a backdrop of inflammatory signaling and immune microenvironment remodeling, representing a key downstream event within the inflammation–immune–tumor interplay network.

A hallmark of EMT is the loss of intercellular adhesion coupled with increased cell motility, typically characterized by downregulation of epithelial markers (e.g., E-cadherin) and upregulation of mesenchymal markers (e.g., N-cadherin, Vimentin). E-cadherin, a central molecule maintaining epithelial polarity and intercellular junctions, is crucial for EMT initiation. Loss of E-cadherin function may result from germline or somatic mutations, promoter hypermethylation, proteolytic cleavage, or transcriptional repression, with promoter hypermethylation being particularly common in cancer cells (45). Hypermethylation recruits methyl-binding proteins and histone-modifying enzymes, establishing a transcriptionally repressive chromatin state that stably silences E-cadherin expression, providing molecular plasticity for tumor cell survival and dissemination in adverse microenvironments. Activation of EMT-related transcription factors, such as Snail, Slug, ZEB1, and Twist, is a core event in this process. These factors repress E-cadherin transcription and are typically activated by growth factors including TGF-β, EGF, and FGF, which may be derived from tumor-associated stromal cells (46). The TGF-β signaling pathway is widely recognized as a major EMT regulator: upon receptor engagement, SMAD2/SMAD4 are activated, upregulating E-cadherin repressors (Snail, Slug, ZEB1, Twist) and promoting expression of mesenchymal markers (N-cadherin, Vimentin, Fibronectin), remodeling the cytoskeleton and enhancing migratory and invasive capacity (47).

Microbial dysbiosis drives EMT and dysregulates cell death through multiple mechanisms. Ye T.T. et al. at Nanchang University reported that OSCC tissues exhibit significant downregulation of E-cadherin and upregulation of N-cadherin, Vimentin, and Snail, indicating EMT activation. Concurrently, NEK2 overexpression correlates with EMT progression, enhancing OSCC invasiveness (48). NEK2, a serine/threonine kinase involved in centrosome separation and chromosomal stability, promotes Snail and Slug expression via Wnt/β-catenin signaling, forming a NEK2–EMT axis that facilitates acquisition of mesenchymal phenotypes. This suggests a novel mechanism by which dysbiosis indirectly activates EMT through modulation of host cell cycle–related gene expression.

Chronic dysbiosis-induced metabolites also contribute to EMT activation. The team at the Affiliated Stomatological Hospital of Chongqing Medical University found that Pseudomonas enrichment under chronic stress generates tryptophan-derived kynurenine, activating the aryl hydrocarbon receptor (AhR) pathway. Kynurenine promotes CD8^+^ T cell exhaustion and upregulates EMT-related transcription factors (Snail, Slug), enhancing tumor invasiveness (49). Kynurenine, produced via indoleamine 2,3-dioxygenase under inflammatory and stress conditions, binds AhR, translocates to the nucleus, and activates downstream gene transcription. This induces inhibitory receptor expression (PD-1, CTLA-4) in CD8^+^ T cells, causing functional exhaustion, while directly upregulating Snail and Slug in epithelial cells, driving EMT. The kynurenine–AhR axis thus represents a critical link connecting microbial metabolism, immune suppression, and tumor invasion.

Additionally, periodontal pathogens such as *Porphyromonas gingivalis* (Pg) can activate the RIP1/RIP3/MLKL pathway to induce necroptosis while inhibiting canonical caspase-dependent apoptosis, sustaining inflammation and promoting EMT (50). Necroptosis, a programmed necrotic form of cell death, disrupts plasma membrane integrity and releases damage-associated molecular patterns (DAMPs), such as HMGB1 and IL-33, activating surrounding immune cells and creating a pro-inflammatory microenvironment. Pg virulence factors, including gingipains, trigger RIP1/RIP3 phosphorylation and necrosome formation, leading to MLKL-mediated membrane permeabilization and cellular content release. Simultaneously, Pg-mediated inhibition of caspase-8 shifts cell death from immunologically silent apoptosis to immunostimulatory necroptosis. DAMPs released from necrotic cell death can further amplify inflammatory signaling and enhance the secretion of proinflammatory cytokines, thereby establishing a sustained inflammation–EMT positive feedback loop.

In summary, microbial dysbiosis drives EMT in OSCC through multidimensional, synergistic mechanisms, representing an extension of chronic inflammation–mediated malignant transformation. Dysbiosis modulates EMT transcription factors directly via metabolites (e.g., kynurenine), indirectly activates EMT by reshaping cell death programs (switching apoptosis to necroptosis) and amplifying inflammation, and regulates host genes (e.g., NEK2), forming a multilayered regulatory network that collectively promotes OSCC invasion and metastasis.

### 3.5 Role of bacterial metabolites

The oral microbiome exerts a critical role in oral tumorigenesis through its metabolites, which act as key effector molecules mediating host–microbe interactions. Bacterial-derived metabolites serve as “molecular messengers,” capable of directly penetrating the epithelial barrier to regulate oral mucosal epithelial cell proliferation, apoptosis, differentiation, and other biological functions. Additionally, they can remodel the tumor immune microenvironment, interfere with immune cell responses, and disrupt anti-tumor immune homeostasis, thereby creating favorable conditions for tumor cell survival, proliferation, invasion, and metastasis. This establishes a cooperative oncogenic network of “oral microbiome–metabolites–host cells–immune microenvironment,” highlighting bacterial metabolites as indispensable contributors to OSCC pathogenesis. This network underscores that microbial dysbiosis is not merely a localized event, but engages in continuous molecular “dialogue“ with the host via metabolites, ultimately reshaping tissue homeostasis and promoting malignant transformation.

Bacterial metabolites are diverse and act through multiple mechanisms, spanning gene mutation, immune modulation, and beyond. Lipopolysaccharide (LPS), a major component of Gram-negative bacterial outer membranes, can stimulate pro-inflammatory cytokine expression and disrupt epithelial barriers, accelerating tumorigenesis. In the oral tumor microenvironment, LPS from Gram-negative bacteria such as *Porphyromonas gingivalis* activates the MyD88/NF-κB signaling pathway via Toll-like receptor 4 (TLR4), This process is closely associated with the aforementioned NF-κB signaling axis, leading to upregulation of proinflammatory cytokines and disruption of the epithelial barrier (51).

Furthermore, metabolites such as butyrate and acetaldehyde produced by Gram-negative bacteria can induce nucleotide mutations and promote carcinogenesis. Butyrate, a principal short-chain fatty acid, exhibits histone deacetylase inhibitory activity at physiological concentrations, modulating gene expression via chromatin remodeling; however, at high concentrations, it induces DNA damage and cell cycle arrest. Kurita-Ochiai et al. reported that under p53-deficient conditions, butyrate can induce apoptosis in T and B lymphocytes (52–54). Mechanistically, p53 loss reduces DNA damage repair capacity and eliminates key apoptotic checkpoints, allowing butyrate to activate mitochondrial pathways (Bax activation, cytochrome c release) or death receptor pathways, upregulating caspase-3 activity to selectively eliminate immune cells. This immunocyte apoptosis diminishes anti-tumor immunity within the tumor microenvironment, reducing immune pressure and enabling tumor cells to acquire invasive phenotypes, thus promoting angiogenesis, local infiltration, and distant metastasis (55, 56). Notably, butyrate’s effects are concentration- and tissue-dependent; in the gut, butyrate is generally anti-inflammatory and barrier-protective, whereas in the oral microenvironment, due to distinct microbial composition and metabolic profiles, it may exert pro-inflammatory and pro-tumorigenic effects. This “environment-dependent functional switch” is pivotal to understanding the pathogenic mechanisms of oral bacterial metabolites.

Moreover, myeloid-derived suppressor cells (MDSCs) are closely associated with tumor metastasis and are abundant in OSCC lesions, where they suppress T cell activation. MDSCs, a heterogeneous population of immature myeloid cells, deplete L-arginine via high expression of arginase-1, inducible nitric oxide synthase, and reactive oxygen species, impairing T cell receptor signaling, while promoting regulatory T cell expansion to establish an immunosuppressive network. *P. gingivalis* promotes MDSC expansion within OSCC lesions through cytokine- and interleukin-mediated signaling, further reinforcing tumor immunosuppression (54). Through metabolites such as LPS, butyrate, and acetaldehyde, *P. gingivalis* simultaneously induces epithelial malignant transformation, selectively depletes immune cells, and amplifies MDSC-mediated immunosuppression. This multifaceted action exemplifies the “multi-target synergistic oncogenic effect” of bacterial metabolites in driving OSCC initiation and progression.

### 3.6 Pathogenic microenvironment and immune evasion mediated by oral biofilms

Oral microorganisms predominantly exist in the host as biofilms rather than in a planktonic state. This three-dimensional structure, embedded within an extracellular polymeric substance (EPS) matrix, provides a physical barrier that enables microbes to resist host immune clearance and antimicrobial agents (57). The EPS matrix mainly consists of bacterial extracellular polysaccharides, proteins, lipids, and extracellular DNA, forming a crosslinked network that confers structural stability while restricting the penetration of antimicrobial compounds and immune effectors such as antibodies and complement. Within biofilms, bacteria communicate through quorum sensing systems, coordinating virulence gene expression and matrix remodeling, thereby enhancing their adaptability to environmental stress. During the malignant transformation of OLP to OSCC, biofilms play several pathogenic roles:

First, biofilm architecture establishes a local hypoxic and acidic microenvironment. The dense metabolic activity of bacteria within the biofilm consumes large amounts of oxygen, while the EPS matrix limits oxygen diffusion, resulting in sustained hypoxia in the deeper biofilm layers. Concurrently, fermentation metabolites such as lactic acid and acetic acid accumulate in the matrix, lowering local pH. This disruption of ecological balance favors colonization and proliferation of anaerobic pathogens, such as *Porphyromonas gingivalis* and *Fusobacterium nucleatum*, and supports biofilm maturation. Hypoxia-inducible factor-1α stabilization may induce metabolic reprogramming in epithelial cells (e.g., enhanced aerobic glycolysis) and activate pro-tumor signaling pathways, and activates inflammation-related signaling pathways such as NF-κB and STAT3, thereby promoting the expression of matrix metalloproteinases and vascular endothelial growth factor, further remodeling the tumor microenvironment and enhancing tumor invasiveness (58, 59).

Second, biofilms act as persistent “antigen reservoirs,” continuously disrupting the oral epithelial barrier, invading the lamina propria, and releasing carcinogenic metabolites and virulence factors such as LPS. These stimuli induce epithelial cells to produce pro-inflammatory cytokines, perpetuating chronic inflammation. The three-dimensional biofilm structure physically resists host immune clearance, while bacteria and their products (e.g., LPS, peptidoglycan, flagellin, nucleic acids) shed from the biofilm surface or are released via outer membrane vesicles. These factors penetrate the compromised epithelial barrier, activating pattern recognition receptors (PRRs) on epithelial and lamina propria immune cells, triggering a cascade of pro-inflammatory cytokines including IL-1β, IL-6, TNF-α, and IL-8. This persistent, low-dose antigenic stimulation shifts inflammation from an acute to a chronic state, forming a “dysbiosis–barrier disruption–inflammation amplification” positive feedback loop, which is a key driver of OLP malignant transformation (57, 60).

Finally, the physical barrier of biofilms and interactions among specific microbial taxa impede immune cell infiltration and cytotoxic function. The EPS matrix not only physically obstructs neutrophils, macrophages, and T cells from penetrating the biofilm but also interacts with immune cell surface receptors via surface polysaccharides, inducing immune cell apoptosis or functional exhaustion. Pathogens such as *F. nucleatum* and *P. gingivalis* interact extensively with macrophages within biofilms and the tumor microenvironment. Through Fap2-mediated recognition of tumor cell surface polysaccharides, these bacteria adhere to host cells and release metabolites (e.g., butyrate, hydrogen sulfide) that drive macrophage polarization toward the M2 phenotype, promoting the secretion of immunosuppressive cytokines such as IL-10 and TGF-β, thereby suppressing CD8^+^ T cell infiltration and cytotoxicity. Moreover, the intratumoral microbiome serves as an “immune sanctuary,” activating myeloid-derived suppressor cells, inducing regulatory T cell expansion, and upregulating immune checkpoint molecules (e.g., PD-L1), thereby facilitating tumor immune evasion and disease progression (61, 62).

In summary, biofilms are not merely a bacterial survival form but serve as a structural and functional foundation for maintaining chronic infection and propagating carcinogenic signaling during OLP malignant transformation. Thus, oral biofilms not only sustain chronic infection but also collaboratively shape a pro-tumor microenvironment by continuously providing inflammatory stimuli, immunosuppressive signals, and metabolic products, thereby driving the malignant transformation of OPMDs into OSCC.

### 3.7 Beyond bacteria: potential roles of fungi and viruses in cross-kingdom carcinogenesis

Although current research has predominantly focused on bacteria, fungi and viruses also play important roles in oral carcinogenesis. These microorganisms engage in complex cross-kingdom dysbiosis, molecular interactions, and synergistic carcinogenic mechanisms with bacteria (63, 64).

Regarding fungi, *Candida albicans* is the most prevalent opportunistic fungal pathogen in patients with oral lichen planus (OLP). Large cohort studies have demonstrated that a high burden of *C. albicans* is an independent risk factor for malignant transformation of oral potentially malignant disorders (OPMDs), and its infection may significantly shorten the latency period from OLP to oral squamous cell carcinoma (OSCC) (65). Mechanistically, *C. albicans* can exacerbate mucosal damage by inducing T helper 17 (Th17)-mediated inflammatory responses, and it possesses strong biofilm-forming capacity. Moreover, its metabolic products, including acetaldehyde and nitrosamines, are potent carcinogens that can directly induce DNA damage in epithelial cells and enhance oxidative stress, thereby promoting malignant progression (66, 67). Furthermore, *C. albicans* often coexists with oral streptococci in cross-kingdom biofilms, further reshaping a highly dynamic and complex tumor microenvironment and amplifying the overall pathogenic potential of the microbial community (63).

In terms of viruses, human papillomavirus (HPV), particularly high-risk types 16 and 18, is a recognized etiological factor for oropharyngeal cancers. Although the precise role of HPV in OLP malignant transformation remains debated, epidemiological evidence indicates that alterations in oral microbial diversity are closely associated with high-risk HPV infection (68). Dysbiosis-induced disruption of the mucosal barrier may facilitate viral entry and persistence (69). Additionally, bacteria-induced chronic inflammatory microenvironments—such as co-activation of NF-κB and STAT3 pathways—may interfere with host immune responses, promoting viral immune evasion. This bacterial–viral synergy can further compromise tumor suppressor pathways, including p53 and retinoblastoma protein (Rb), accelerating cell cycle dysregulation and carcinogenesis (63, 69).

In summary, the carcinogenic mechanisms of the oral microbiome constitute a complex network involving bacteria, fungi, and viruses. Future research should focus on elucidating the molecular interactions and specific contributions of non-bacterial microbes in the malignant transformation of OLP.

## 4 Discussion

Integrating current evidence, microbiota-associated carcinogenic mechanisms—including chronic inflammation, oxidative stress, epithelial–mesenchymal transition (EMT), and immune evasion—do not act in isolation, but collaboratively contribute to the dynamic remodeling of the tumor microenvironment in head and neck squamous cell carcinoma (HNSCC). Dysbiosis of the oral microbiota can activate key signaling pathways such as NF-κB and STAT3, thereby modulating inflammatory cytokine networks, immune cell recruitment, and stromal interactions, which play critical roles in tumor initiation and progression. Recent studies emphasize that interventions targeting the tumor microenvironment—such as immune checkpoint blockade, regulation of cancer-associated fibroblasts, and strategies addressing hypoxic niches—may offer novel therapeutic targets and avenues for HNSCC (71). Therefore, integrating microbiota-driven molecular mechanisms with tumor microenvironment-targeted therapies may provide important theoretical guidance for translational research and precision treatment in HNSCC.

### 4.1 Current controversies and knowledge gaps

Regarding experimentally validated causal relationships, several key bacteria and their mechanisms have been confirmed. For instance, *Fusobacterium nucleatum* has been shown *in vitro* to produce hydrogen sulfide (H_2_S), inhibit superoxide dismutase (SOD) activity, and induce excessive reactive oxygen species (ROS) accumulation, resulting in oxidative DNA damage, such as elevated 8-oxo-dG levels (35). *In vivo* animal models further demonstrate that *F. nucleatum* can activate NF-κB and STAT3 signaling, promoting OSCC cell proliferation, invasion, and EMT (50).

In contrast, evidence based solely on associative observations mainly concerns changes in the relative abundance of specific taxa. Clinical cohort studies have reported increased relative abundance of *Prevotella intermedia*, *Treponema denticola*, and *Campylobacter rectus* in OSCC patients (41); however, *in vitro* or *in vivo* studies directly demonstrating that these bacteria induce DNA damage or suppress immune surveillance are still lacking.

Moreover, microbial patterns vary across geographic populations. Fusobacteria abundance and core community composition differ between Asian and Western cohorts, likely influenced by genetic background, diet, or sequencing methodology. *Streptococcus mutans* has been identified as a potential pro-carcinogenic species in Asian studies (10, 41), whereas some Western studies found no association; evidence regarding a protective role of *Bifidobacterium* remains inconclusive.

Most current studies rely on 16S rRNA sequencing, which provides compositional information but cannot resolve functional mechanisms. Applications of metagenomics and metabolomics remain limited. In addition, differences in sampling sites (saliva versus tissue biopsy), lack of standardized experimental design, and insufficient control of confounding factors such as smoking and alcohol consumption further constrain causal inference (72).

Therefore, while current evidence primarily supports an association between the oral microbiota and oral cancer, establishing direct causality requires additional longitudinal studies and functional validation.

### 4.2 Limitations

It should be acknowledged that this field and the present review face several limitations: methodological heterogeneity (sequencing platform, sampling site, population differences) may affect result comparability; cross-sectional designs cannot reveal temporal or causal relationships; research has largely focused on bacteria, with limited exploration of viral and multi-kingdom microbial interactions; standardized diagnostic criteria for the oral microbiota are lacking. These represent critical challenges for future translational research.

In summary, dynamic changes in the oral microbiota hold promise not only as early potential biomarkers for malignant transformation of OLP, improving early diagnostic capability, but also as targets for prevention and intervention. With the rapid development of multi-omics technologies, future research should integrate genomics, transcriptomics, and metabolomics to systematically elucidate the complex host–microbiota network. Large-scale longitudinal cohort studies will enable tracking of microbiota evolution and its causal relationship with disease progression, facilitating in-depth exploration of underlying molecular mechanisms. Such efforts may ultimately support the development of precision strategies targeting oral microecology, intervene in disease progression, improve clinical outcomes, and open new avenues for the management of oral potentially malignant disorders.

## 5 Conclusion

The oral microbiome participates in the malignant transformation of OLP to oral squamous cell carcinoma (OSCC) through multiple mechanisms, playing key roles in inflammation regulation, immune modulation, and metabolic homeostasis. Dysbiosis may exacerbate local chronic inflammation, promote immune evasion, and disrupt normal cellular metabolism, thereby facilitating malignant progression. Thus, an in-depth understanding of the specific mechanisms by which the oral microbiome contributes to this transformation is critical for elucidating the microbial ecological basis of oral carcinogenesis. Moreover, microbiome-based interventions provide new directions for clinical prevention and treatment. Future studies integrating multi-omics approaches with longitudinal clinical investigations will allow for a systematic assessment of microbiome dynamics and host interactions, ultimately facilitating the development of precise, microbiome-targeted intervention strategies to improve the management and prognosis of OPMDs.
